# Longitudinal [^18^F]GE-180 PET Imaging Facilitates In Vivo Monitoring of TSPO Expression in the GL261 Glioblastoma Mouse Model

**DOI:** 10.3390/biomedicines10040738

**Published:** 2022-03-22

**Authors:** Adrien Holzgreve, Dennis Pötter, Matthias Brendel, Michael Orth, Lorraine Weidner, Lukas Gold, Maximilian A. Kirchner, Laura M. Bartos, Lena M. Unterrainer, Marcus Unterrainer, Katja Steiger, Louisa von Baumgarten, Maximilian Niyazi, Claus Belka, Peter Bartenstein, Markus J. Riemenschneider, Kirsten Lauber, Nathalie L. Albert

**Affiliations:** 1 Department of Nuclear Medicine, University Hospital, Ludwig Maximilian University of Munich (LMU Munich), 81377 Munich, Germany; adrien.holzgreve@med.lmu.de (A.H.); dennis.poetter@med.uni-muenchen.de (D.P.); matthias.brendel@med.uni-muenchen.de (M.B.); lukas.gold@med.uni-muenchen.de (L.G.); maximilian.kirchner@med.uni-muenchen.de (M.A.K.); laura.bartos@med.uni-muenchen.de (L.M.B.); lena.unterrainer@med.uni-muenchen.de (L.M.U.); peter.bartenstein@med.uni-muenchen.de (P.B.); 2 Department of Radiation Oncology, University Hospital, Ludwig Maximilian University of Munich (LMU Munich), 81377 Munich, Germany; michael.orth@med.uni-muenchen.de (M.O.); maximilian.niyazi@med.uni-muenchen.de (M.N.); claus.belka@med.uni-muenchen.de (C.B.); kirsten.lauber@med.uni-muenchen.de (K.L.); 3 Department of Neuropathology, Regensburg University Hospital, 93053 Regensburg, Germany; lorraine.weidner@klinik.uni-regensburg.de (L.W.); markus.riemenschneider@klinik.uni-regensburg.de (M.J.R.); 4 Department of Radiology, University Hospital, Ludwig Maximilian University of Munich (LMU Munich), 81377 Munich, Germany; marcus.unterrainer@med.uni-muenchen.de; 5 German Cancer Consortium (DKTK), Partner Site Munich, German Cancer Research Center (DKFZ), 69120 Heidelberg, Germany; katja.steiger@tum.de (K.S.); louisa.vonbaumgarten@med.uni-muenchen.de (L.v.B.); 6 Institute of Pathology, TUM School of Medicine, Technical University of Munich, 81675 Munich, Germany; 7 Department of Neurosurgery, University Hospital, Ludwig Maximilian University of Munich (LMU Munich), 81377 Munich, Germany

**Keywords:** [^18^F]GE-180 PET, 18 kDa translocator protein (TSPO), GL261, glioblastoma, ex vivo and in vitro autoradiography

## Abstract

The 18 kDa translocator protein (TSPO) is increasingly recognized as an interesting target for the imaging of glioblastoma (GBM). Here, we investigated TSPO PET imaging and autoradiography in the frequently used GL261 glioblastoma mouse model and aimed to generate insights into the temporal evolution of TSPO radioligand uptake in glioblastoma in a preclinical setting. We performed a longitudinal [^18^F]GE-180 PET imaging study from day 4 to 14 post inoculation in the orthotopic syngeneic GL261 GBM mouse model (*n* = 21 GBM mice, *n* = 3 sham mice). Contrast-enhanced computed tomography (CT) was performed at the day of the final PET scan (±1 day). [^18^F]GE-180 autoradiography was performed on day 7, 11 and 14 (ex vivo: *n* = 13 GBM mice, *n* = 1 sham mouse; in vitro: *n* = 21 GBM mice; *n* = 2 sham mice). Brain sections were also used for hematoxylin and eosin (H&E) staining and TSPO immunohistochemistry. [^18^F]GE-180 uptake in PET was elevated at the site of inoculation in GBM mice as compared to sham mice at day 11 and later (at day 14, TBR_max_ +27% compared to sham mice, *p* = 0.001). In GBM mice, [^18^F]GE-180 uptake continuously increased over time, e.g., at day 11, mean TBR_max_ +16% compared to day 4, *p* = 0.011. [^18^F]GE-180 uptake as depicted by PET was in all mice co-localized with contrast-enhancement in CT and tissue-based findings. [^18^F]GE-180 ex vivo and in vitro autoradiography showed highly congruent tracer distribution (*r* = 0.99, *n* = 13, *p* < 0.001). In conclusion, [^18^F]GE-180 PET imaging facilitates non-invasive in vivo monitoring of TSPO expression in the GL261 GBM mouse model. [^18^F]GE-180 in vitro autoradiography is a convenient surrogate for ex vivo autoradiography, allowing for straightforward identification of suitable models and scan time-points on previously generated tissue sections.

## 1. Introduction

The 18 kDa translocator protein (TSPO) is increasingly recognized as an interesting target for the study of glioblastoma (GBM), the most common and aggressive primary malignant brain tumor in adults with a five-year survival rate of only 7.2% [1]. Effective treatment options remain limited, redefined tumor classification and improved chemotherapy regimens resulted in a median overall survival of up to 4 years depending on the molecular subgroup; however, most glioblastoma patients die earlier [2,3]. Originally mainly subject to research in neuroinflammation [4,5,6], TSPO appears to assume a pivotal role in resistance to apoptosis, invasiveness, and proliferation in GBM [7]. Eventually, TSPO—also known as peripheral-type benzodiazepine receptor (PBR)—may be an important functional stakeholder in tumorigenesis and treatment resistance of GBM.

Positron emission tomography (PET) has gained recognition in neuro-oncology as a valuable molecular imaging tool especially using the radiolabeled amino acid analog *O*-(2-[^18^F]fluoroethyl)-L-tyrosine ([^18^F]FET) [8,9,10]. In the wake of its recognition as a functionally relevant target in GBM, increasing experience has recently been obtained with glioma imaging directed against TSPO, including the use of PET tracers such as [^18^F]GE-180, [^18^F]DPA-714, [^18^F]PBR111 and [^18^F]VC701 [7,11,12]. While a few preliminary studies have pointed to a potential clinical benefit of TSPO PET in glioblastoma patients attributed to additional information as compared to established imaging modalities [13,14], the potential of TSPO imaging in preclinical studies is currently a thriving and promising field in order to better understand the glioma microenvironment [15]. Although most of the recent human data on TSPO PET imaging in GBM has been achieved using the tracer [^18^F]GE-180, until now it has not experienced as much use in preclinical GBM PET imaging studies [13,14,16,17,18,19,20,21].

Here, we investigated the feasibility of TSPO PET imaging using the high-affinity TSPO ligand [^18^F]GE-180 for the first time in GBM in the preclinical setting, and we hypothesized that longitudinal PET imaging facilitates in vivo monitoring of TSPO expression over time. To this end, we performed a longitudinal PET study in the syngeneic GL261 GBM mouse model, being one of the most frequently used GBM mouse models. TSPO PET imaging was correlated with contrast-enhanced computed tomography (CT), and in vivo findings were verified by in vitro methods including [^18^F]GE-180 autoradiography and TSPO immunohistochemistry (IHC). Furthermore, we performed a head-to-head comparison of ex vivo and in vitro [^18^F]GE-180 autoradiography with regard to regional tracer distribution in the brain of GL261-bearing mice.

## 2. Materials and Methods

### 2.1. Study Design

All experiments were performed in compliance with the National Guidelines for Animal Protection in Germany with approval of the local care committee of the Government of Oberbayern (Regierung von Oberbayern) and overseen by a veterinarian. In total, 24 female C57BL/6 mice, 10–12 weeks old, were delivered by Charles River (Sulzfeld, Germany) and acclimated for one week. Animals were housed in a temperature- and humidity-controlled environment (25 °C and 65% rH, respectively) with a 12 h light–dark cycle, with free access to food and water. At day 0, mice were orthotopically inoculated either with GL261 (GBM mice) or with saline for control (sham mice).

One cohort of mice (*n* = 9 GBM mice, *n* = 3 sham mice) was scanned longitudinally on up to four time-points post inoculation (day 4, 7, 11 and 14; *n* = 40 TSPO PET scans), and single mice were sacrificed for tissue-based analyses earlier than day 14. To increase sample size for tissue-based analysis, one other cohort (*n* = 12 GBM mice) was scanned cross-sectionally on different single time-points post inoculation (day 7, 11 or 14; *n* = 12 TSPO PET scans). Contrast-enhanced computed tomography (CT) was performed at the day of the final PET scan (±1 day). Subsequently to PET scans, mice received intracardiac perfusion with 4% paraformaldehyde (PFA) to fix the brain tissue for ex vivo analysis. Ex vivo TSPO autoradiography (*n* = 13 GBM mice; *n* = 1 sham mouse) and in vitro TSPO autoradiography (*n* = 21 GBM mice; *n* = 2 sham mice) were performed on day 7, 11 and 14. Brain sections were also used for hematoxylin and eosin (H&E) staining (*n* = 17 GBM mice; *n* = 2 sham mice) and TSPO IHC (*n* = 8 GBM mice, *n* = 1 sham mouse) afterwards.

First, TSPO radioligand uptake in PET was quantified at different time points comparing GBM mice and sham mice. Second, uptake changes over time were analyzed. Eventually, the location and extent of uptake in PET were correlated to CT and in vitro findings. Further, ex vivo and in vitro autoradiography performed on same brain slices were directly compared with regard to tracer uptake patterns.

**Figure 1:**
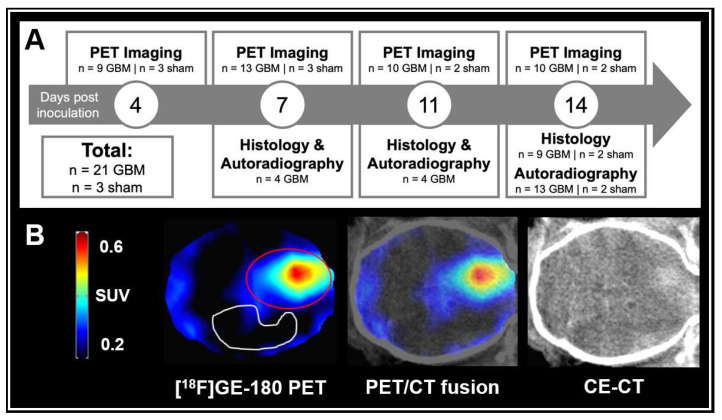
(**A**) Study overview. *n* = 10 GBM mice and *n* = 2 sham mice completed longitudinal imaging until day 14 post inoculation. (**B**) GBM mouse at day 14 after tumor cell inoculation. [^18^F]GE-180 PET (left), a digital PET/CT fusion (middle) and CE-CT (right) are shown in axial plane. The [^18^F]GE-180 PET images were masked according to whole brain in CE-CT. Volume-of-interests (VOIs) used for uptake quantification are shown: Red line = 36 mm^3^ VOI at the site of inoculation. White line = 53 mm^3^ VOI in the tumor-free contralateral hemisphere serving as background.

An overview of the study is provided in Figure 1A.

### 2.2. Animal Model

Intracranial implantation of tumor cells and intracranial saline injection were performed as previously published with slight modifications [22]. In brief, mice received a pre-medication of 200 μg/g body weight metamizole (WDT, Garbsen, Germany) 2 h prior the surgery. Anesthesia consisted of intraperitoneal injection of 100 μg/g ketamine and 10 μg/g xylazine (both from WDT, Garbsen, Germany). The mouse head was fixed on a stereotaxic frame heated at 37 °C (David Kopf Instruments, Tujunga, CA, USA). After skin incision, the burr hole was placed 1.5 mm lateral (right) and 1 mm anterior to the bregma (23G/21G microlances, BD Biosciences, Heidelberg, Germany). Either 1 × 10^4^ GL261 tumor cells in 1 µL of saline or 1 µL of saline alone were injected into the right striatum at 1.5–3.0 mm depth using a stereotactically guided glass syringe (22G, Hamilton Bonaduz, Bonaduz, Switzerland). After skin closure with Ethibond Excel 5–0 suture material (Ethicon, Norderstedt, Germany), the mice were kept under surveillance on a heated pad until full recovery.

### 2.3. TSPO PET

All mice received [^18^F]GE-180 PET scans. Each mouse received bolus injection of 12.5 ± 2.2 MBq of [^18^F]GE-180 in 150 µL of saline into the tail vein [23]. Radiosynthesis of [^18^F]GE-180 was performed as previously described [24] with slight modifications [25], eventually resulting in a radiochemical purity >98% and a specific activity of 1400 ± 500 GBq/μmol. Anesthesia was performed with isoflurane 2% delivered via mask at 3.5 L/min in oxygen. Four mice were placed simultaneously in the tomograph (Siemens Inveon PET, Siemens Healthineers, Erlangen, Germany). Emission recording was performed for the interval 60–90 min post injection (p. i.) followed by a 15 min transmission scan using a rotating [^57^Co] point source as previously described [25]. The PET images were reconstructed as previously published [26]: A 3D ordered-subset expectation maximization (OSEM) with 4 iterations and 12 subsets was performed and succeeded by a maximum a posteriori (MAP) algorithm with 32 iterations [26]. An attenuation correction was performed using the transmission scan obtained with the rotating ^57^Co point source. The scattering contributions were estimated using the transmission data set and simulations for a limited number of scattering points in the object. We applied a decay correction for ^18^F. The zoom factor was 1.0, the matrix was 256 × 256 × 159. The final voxel dimension was 0.78 mm × 0.78 mm × 0.80 mm [27].

Myocardial tracer uptake was used as normalization procedure as previously established [26].

For quantitative analysis, a manually generated uniform tumor VOI with a volume of 36 mm^3^ was created based on an average image of all scans performed. It was double-checked for all mice individually that the visually increased uptake at the site of inoculation was comprised by the uniform tumor VOI. Another manually generated VOI with a volume of 53 mm^3^ in the tumor-free contralateral hemisphere was set as background. For VOI definition, see Figure 1B.

### 2.4. TSPO Autoradiography

Ex vivo autoradiography was performed directly after the final [^18^F]GE-180 PET scan as shown before [27]: After intracardiac perfusion with PBS and subsequently with 4% PFA (circulation stop at 105 min p. i.), the brains were cooled down for a maximum of 5 min at −80 °C. After ca. 10 more min at −20 °C, brains were horizontally cut in 16 µm sections for autoradiography or in 3 µm sections for immunohistochemistry using a Leica CM1510 Cryostat (Leica Microsystems, Nussloch, Germany).

In vitro autoradiography was performed on 16 µm horizontal brain cryosections: After pre-incubation with binding buffer (Tris-HCl 50 mM, pH 7.4) and drying, the sections were incubated for 60 min with 0.06 MBq/mL of [^18^F]GE-180 in binding buffer at room temperature. After incubation, sections were washed twice by immersion in ice-cold buffer solution, dried and placed on imaging plates for 24 h [28]. The obtained data was analyzed with AIDA image analyzing software (version 4.50; Elysia-raytest GmbH, Straubenhardt, Germany). A manually drawn region of interest (ROI) was placed in the contralateral tumor-free background and sections were scaled to mean background activity; prior to quantification, the photo plate background was subtracted (in analogy to [29]). Due to single processing artifacts (e.g., freezing damage), an automated target region segmentation for all sections was discarded, and target ROIs were created via manually adjusted hot-seed function [27]. Area tissue and mean radioactivity concentrations per area tissue were measured. The ROIs were used for volumetric approximation according to the Cavalieri method [30] on every 24th section.

### 2.5. TSPO Immunohistochemistry and Hematoxylin and Eosin (H&E) Staining

Cryo-conserved brain tissue was sampled from sham or tumor-cell-inoculated mice for immunohistochemical staining. TSPO staining was performed following a standard protocol [31]. After cutting, a positive control was deparaffinized, and after short thawing, the sample slices as well as the positive control underwent Tris/EDTA buffer antigen retrieval and blocking. Slices were then incubated with the primary antibody for 1 h. As primary antibody, we used the anti-PBR antibody [EPR5384] (from Abcam, Berlin, Germany) at 1:100 dilution. The EnVision^TM^+ Dual Link System-HRP (Dako by Agilent Technologies, Santa Clara, CA, USA) was utilized for detection of antibody binding according to the manufacturer’s protocol (Kit K4065, https://www.agilent.com/cs/library/packageinsert/public/PD04048EN_02.pdf, accessed on 8 March 2022). Slices were then counterstained with hematoxylin, dehydrated and coverslipped.

A further subset of the prepared brain sections was processed by hematoxylin and eosin (H&E) staining for histopathological analysis, after being temporarily stored at −80 °C. Photographs of the tumors were taken with a Primo Star/Axiocam 105 color microscope (Zeiss, Jena, Germany). ROIs were created with ImageJ (National Institutes of Health, Bethesda, MD, USA) and VOIs were estimated as described above.

Autoradiographies and consecutive H&E staining were conducted on the exact same slices while immediate adjacent slices were used for immunohistochemistry.

### 2.6. Contrast-Enhanced Computed Tomography (CE-CT)

For the purpose of morphological correlation, a subgroup of mice underwent CE-CT scans as previously described [22]. Mice were anaesthetized with isoflurane 2% delivered via mask at 3.5 L/min in oxygen and received an intravenous bolus injection of 300 µL imeron-300 (equivalent to 90 mg iodine, Bracco Imaging, Konstanz, Germany) 3 min prior to CT acquisition for contrast enhancement. The CT scan was performed using a small animal radiation research platform (SARRP, Xstrahl, Camberley, UK) for the first cohort of mice and a Molecubes X-Cube (Molecubes, Belgium) for the second cohort of mice.

### 2.7. Statistical Analysis

Statistical analysis was performed using IBM SPSS Statistics (version 25; SPSS, IBM, Armonk, NY, USA). Group comparisons of VOI-based PET results between pooled sham mice and GBM mice and at different days post inoculation were tested using a one-way-ANOVA with subsequent Tukey post hoc test for multiple comparisons. Similarity of volumes in ex vivo and in vitro autoradiography was expressed with Pearson’s correlation coefficient. A threshold of *p* < 0.05 was considered to be significant for rejection of the null hypothesis.

## 3. Results

### 3.1. TSPO PET

**Figure 2:**
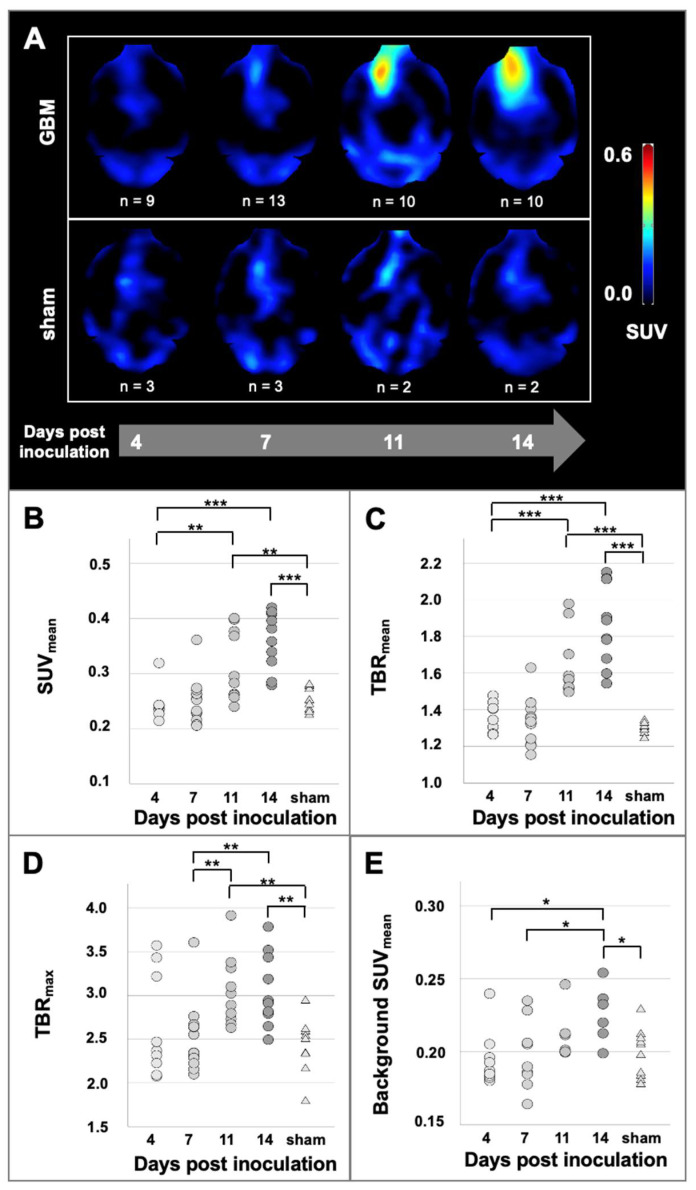
(**A**) Longitudinal [^18^F]GE-180 PET images of GBM mice and sham mice. The [^18^F]GE-180 PET images were masked according to whole brain in CE-CT. Quantification of [^18^F]GE-180 uptake in PET at the inoculation site (**B**–**D**) and at the background in the contralateral hemisphere (**E**). The GBM mice are grouped according to their scan date (*n* = 9 at day 4, *n* = 13 at day 7, *n* = 10 at day 11 and *n* = 10 at day 14 post inoculation). The sham mice are pooled (*n* = 10). Significant differences between groups are marked by *** *p* < 0.001; ** *p* < 0.01; * *p* < 0.05 (ANOVA followed by Tukey post hoc test).

A total of 52 [^18^F]GE-180 PET scans was carried out. All GBM mice presented increased [^18^F]GE-180 uptake at the inoculation site. The increased uptake in PET was co-localized with contrast-enhancement in CT at the site of inoculation (see Figure 1B). The [^18^F]GE-180 uptake in PET visually increased over time both by signal intensity and extent. [^18^F]GE-180 uptake was also visually observed in sham mice along the inoculation scar. In contrast to GBM mice, the extent of [^18^F]GE-180 uptake in sham mice did not increase over time (see Figure 2A).

Using a uniform tumor VOI for quantitative assessment of tracer uptake at the inoculation site, [^18^F]GE-180 uptake steadily increased over time in GBM mice finally mounting to mean SUV_mean_ 0.36 ± 0.05 at day 14 post inoculation (*p* < 0.001, compared to day 4, see Figure 2).

When compared to pooled sham mice, GBM mice showed elevated mean SUV_mean_ on day 11 post inoculation (+26%; *p* = 0.012) and day 14 post inoculation (+44%; *p* < 0.001) at the site of inoculation. Likewise, tumor-to-brain ratios were significantly higher in GBM mice as compared to sham mice on day 11 post inoculation (TBR_mean_ +27%; *p* < 0.001; TBR_max_ +26%; *p* = 0.001) and day 14 post inoculation (TBR_mean_ +44%; *p* < 0.001; TBR_max_ +27%; *p* = 0.001), see Figure 2.

Interestingly, while the background [^18^F]GE-180 uptake at early time points was comparable between GBM mice and sham mice, it slightly but significantly increased over time in GBM mice (mean SUV_mean_ + 8% from day 4 to day 14, *p* = 0.025) and was 10% higher in GBM than in sham animals on day 14 post inoculation (*p* = 0.02).

All TBR and SUV_mean_ values for all mice included in the study are displayed in Figure 2.

Tumor target regions as depicted by [^18^F]GE-180 PET were in all mice co-localized with contrast-enhancement in CT. Tumoral extent and growth were visually confirmed by CE-CT.

### 3.2. TSPO Autoradiography

**Figure 3:**
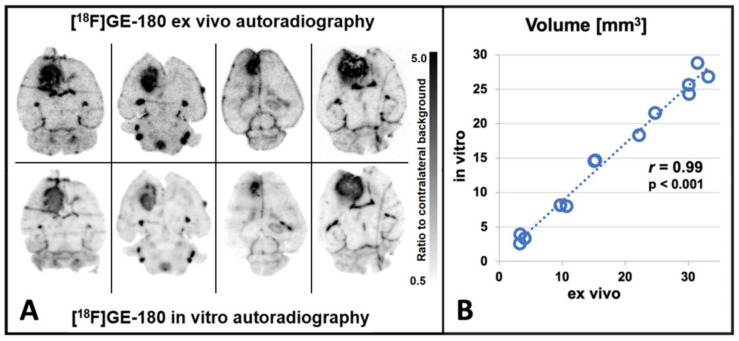
(**A**) [^18^F]GE-180 ex vivo and in vitro autoradiography performed on the exact same brain slices show highly congruent uptake. (**A**) Visual comparison of [^18^F]GE-180 uptake patterns in GBM mice at day 14 post inoculation. (**B**) Quantitative comparison of tracer distribution in GBM mice (Pearson’s *r* = 0.99, *p* < 0.001, *n* = 13).

**Figure 4:**
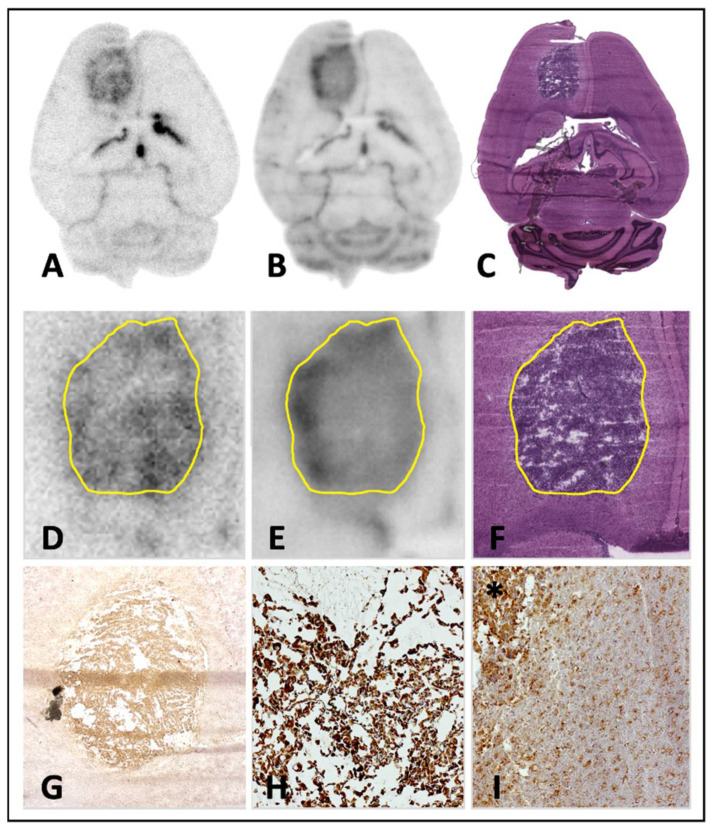
Correlation of [^18^F]GE-180 autoradiography, TSPO immunohistochemistry and histology in axial C57BL/6 mouse brain slices 14 days after inoculation of GL261 cells. (**A**) [^18^F]GE-180 ex vivo autoradiography; ×1. (**B**) [^18^F]GE-180 in vitro autoradiography; ×1. (**C**) Hematoxylin and eosin (H&E) staining; ×1. (**D**) [^18^F]GE-180 ex vivo autoradiography with tumor delineation in H&E staining; ×4. (**E**) [^18^F]GE-180 in vitro autoradiography with tumor delineation in H&E staining; ×4. (**F**) H&E staining; ×4. (**G**)TSPO immunohistochemistry; ×20. (**H**) TSPO immunohistochemistry of the tumor center; ×100. (**I**) TSPO immunohistochemistry of the tumor (*) and the tumor border; ×100.

All GBM mice showed highly elevated [^18^F]GE-180 uptake at the inoculation site. Ex vivo and in vitro autoradiography were compared on the exact same slices (*n* = 13 GBM mice, *n* = 196 slices) and visually showed a highly congruent [^18^F]GE-180 uptake pattern and intensity (see Figure 3A), as previously reported in a single case [16]. A quantitative comparison of volumes in ex vivo and in vitro autoradiography confirmed a high congruency between both methods resulting in a high Pearson’s correlation coefficient of *r* = 0.99 (*n* = 13, *p* < 0.001; see Figure 3B). Furthermore, autoradiography volumes were congruent to TSPO and H&E staining results (see Figure 4).

Mean tumor volume, estimated by in vitro autoradiography, was 1.3 ± 0.7 mm^3^ on day 7 post inoculation, 3.3 ± 0.6 mm^3^ on day 11 post inoculation and 16.1 ± 8.7 mm^3^ on day 14 post inoculation. Sham mice showed signal enhancement consistent with the inoculation scar; however, the enhancement was mostly too small for reliable volumetry.

### 3.3. TSPO Immunohistochemistry and Histology

Immunohistochemical staining confirmed high TSPO expression in the right frontal lobe at the site of inoculation in GBM mice. The extent of TSPO expression in immunohistochemistry was visually congruent with the extent of [^18^F]GE-180 uptake in autoradiography as well as with the tumor borders in H&E staining (see Figure 4) with an accentuated [^18^F]GE-180 uptake and TSPO expression at the tumor margin.

**Figure 5:**
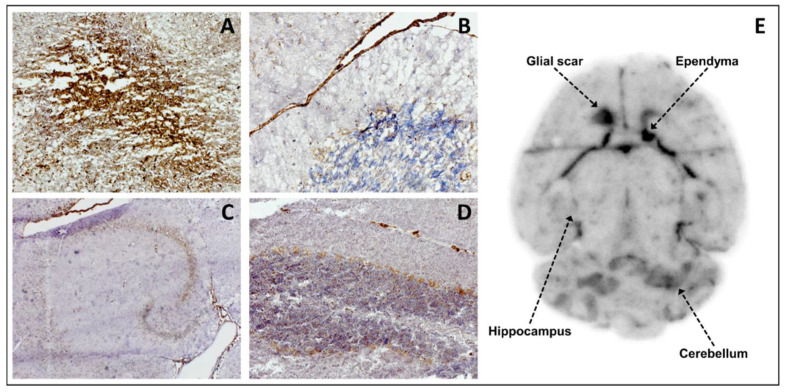
Correlation of [^18^F]GE-180 autoradiography and TSPO immunohistochemistry in axial brain slices of a sham-operated C57BL/6 mouse. (**A**) TSPO staining of the glial scar at the site of the invasive inoculation; ×100. (**B**) TSPO staining of the ependyma; ×200. (**C**) TSPO staining of the hippocampal region; ×40. TSPO expression is found in CA1–CA3 neurons and the dentate gyrus. (**D**) TSPO staining of the cerebellum with a small portion of arachnoid mater; ×100. TSPO expression is detectable in the Purkinje cells of the cerebellum and in the arachnoid cells. (**E**) Anatomical correlation of sites of TSPO expression with [^18^F]GE-180 uptake in in vitro autoradiography.

Moreover, TSPO expression was proven in several brain structures apart from the GL261 tumor such as CA1-CA3 neurons and dentate gyrus in hippocampus, ependyma, cerebellar Purkinje cells, and glial scar of the inoculation process (see Figure 5), which also mirrors PET and autoradiography findings in sham mice (e.g., see Figure 2A).

H&E staining showed spherical tumor growth in the right frontal lobe in all GBM cases. The histologically estimated tumor volume was 0.5 ± 0.1 mm^3^ on day 7 post inoculation and increased up to 16.2 ± 9.9 mm^3^ on day 14 post inoculation.

## 4. Discussion

In this multimodal longitudinal [^18^F]GE-180 PET study we corroborate the translocator protein TSPO as an interesting target for in vivo imaging of glioblastoma, as confirmed by ex vivo autoradiography, in vitro autoradiography and TSPO staining in a preclinical setting.

[^18^F]GE-180 PET and [^18^F]GE-180 autoradiography showed high tracer uptake at the site of inoculation as compared to the contralateral brain hemisphere in the GL261 glioblastoma mouse model, and [^18^F]GE-180 uptake in PET increased over time in GBM mice. Both reader-dependent visual evaluation and objective quantitative analysis provided a reliable differentiation between tumor mice and healthy sham mice from at least day 11 after inoculation. Given the rapidly growing tumor model, the comparatively modest SUV progression of tumor mice may appear to be somewhat underestimated. This may be due in part to the use of a uniform tumor VOI, which in principle includes an excessive amount of non-tumor tissue in the case of small tumors, but also in part to the role of various origins of the TSPO signal, as discussed in more detail below. The in vivo imaging results were supported by ex vivo findings, which showed a high spatial overlap between TSPO staining and [^18^F]GE-180 uptake in high resolution autoradiography. On this occasion, we noted in a large data set of *n* = 196 brain slices, that in vitro autoradiography and ex vivo autoradiography show equal [^18^F]GE-180 uptake patterns and intensity (Pearson’s *r* = 0.99, *p* < 0.001, *n* = 13 GBM mice), which eventually allows for post hoc correlation of TSPO radioligand uptake via in vitro autoradiography with other targets on the very same brain slice (e.g., see Figure 3). This might especially facilitate regional ex vivo co-localization of TSPO expression in the frame of double tracer PET studies, which gain increasing importance also for the understanding of the glioblastoma metabolism and microenvironment [19,32,33].

Sham mice also showed a signal enhancement in the inoculation area, probably due to neuroinflammation, as further elucidated below [33,34]. However, unlike this relatively low uptake related to the traumatic inoculation procedure, the higher tumor-associated [^18^F]GE-180 uptake clearly increased over time both in extent and intensity (see Figure 2A). Quantitative [^18^F]GE-180 PET analysis substantiated significant differences between GBM mice and healthy sham mice from day 11 post inoculation forward using SUV measurements and tumor-to-background ratios (see Figure 2B). Compared to [^18^F]FET in a relatable study setting [27], [^18^F]GE-180 provides a higher tracer uptake at the site of inoculation and excellent tumor-to-background ratios in the early stages of the disease as described in human data as well [19]. As recently shown, longitudinal TSPO PET imaging in glioblastoma in vivo models is an efficient tool to monitor treatment response and delivers different information compared to established tracers [32]. The current study aimed to investigate [^18^F]GE-180 PET imaging in one of the most common syngeneic mouse glioblastoma models, GL261. However, the inclusion of various additional tumor models in in vivo imaging studies will be of interest, as different molecularly defined subtypes of GBM may show different levels of TSPO expression, and TSPO-targeted PET imaging might therefore be useful to non-invasively assess the latter [35,36]. Although [^18^F]GE-180 PET is widely been used in other disease entities such as neuroinflammatory diseases [37], so far, TSPO PET imaging is overall of limited value for extra-cerebral oncologic diseases and has only sporadically been evaluated in other cancer entities than GBM, such as malignant pancreatic lesions [38].

Although [^18^F]GE-180 provides a high tumor-to-background contrast which facilitates intra-cerebral tumor delineation, in vivo tumor volumetry in TSPO PET is hampered by physiological tracer uptake and perfusion-related increased signal in adjacent structures such as Harderian glands, the olfactory epithelium, the skull base and the pituitary gland. We decided to use a reference region in the contralateral hemisphere (see Figure 1A) to consider quantitative variability through individual differences in blood flow and other mouse specific confounders [39,40]. At later time points, we found a 10% higher radiotracer uptake in the contralateral hemisphere in tumor-bearing mice compared to sham mice (*p* = 0.02). This finding might indicate an affection of the hemisphere contralateral to the macroscopic tumor, either being of neuroinflammatory nature, or in the scope of long-range signaling within multicellular networks in glioma, as suggested in recent discoveries on the neuroscience of gliomas [41,42]. Thus, the increased background TSPO upregulation over time in the GL261 model would support the conception of glioblastoma being a disease of the entire brain [43]. However, this finding is limited by a low number of sham cases at later time points and therefore should not be overemphasized.

It is still a matter of debate to what proportion TSPO expression in glioma is related to tumor cells and to inflammatory cells such as glioma-associated microglia/macrophages (GAM). Since both cell types have been shown to overexpress TSPO, the high [^18^F]GE-180 uptake into GL261 tumors in the present study might represent a symbiosis of both [33,44,45]. Further cell types such as activated astrocytes and endothelial cells express TSPO in the context of brain diseases and thus are likely to contribute to a certain degree to the TSPO PET signal in brain tumors [46,47]. Currently ongoing in vivo and in vitro studies, both in humans and rodents, need to provide further clarity on the origin of TSPO radioligand uptake in brain tumors. Here, e.g., longitudinal double tracer PET studies including both [^18^F]FET and [^18^F]GE-180 in glioblastoma mouse models would be helpful to gain further in vivo insights on the inflammatory contribution to the TSPO PET signal. Beyond tumor-associated inflammation, the slightly increased [^18^F]GE-180 uptake at the site of inoculation in sham-operated mice in the current study supports the assumption of an additional inflammatory component solely related to traumatic brain injury, which is in line with previous TSPO PET studies on brain injury mainly in rats [48,49]. A first study has used TSPO PET imaging in glioma using a TSPO knock out (KO) mouse strain, which might also be a promising tool to further decipher the origin of TSPO-related molecular processes in glioblastoma [11]. A major advantage of this approach is that TSPO KO mice should not express endogenous TSPO and therefore represent a “null-background host”. Thus, after tumor cell inoculation, the level of tumor cell-related TSPO expression can be monitored without interfering signal from endogenously TSPO-expressing macrophages, microglia, activated astrocytes or other cells of the host. Specifically, the authors compared the TSPO radioligand uptake in PET from wildtype GL261 glioma in TSPO KO mice with the uptake of wildtype GL261 glioma in wildtype host tissue (TSPO+/+). They found that in TSPO+/+ the signal extended beyond the tumor, whereas in TSPO KO the signal was lower and restricted to the tumor, indicating that in the wildtype situation—as in our study—the TSPO PET signal in the GL261 model is indeed of diverse cellular origin [11]. The authors used magnetic resonance imaging (MRI) as a reference modality for tumor extent. However, [^18^F]FET PET is known to depict vital brain tumor tissue beyond tumor extent in MRI [50]; therefore, it would also be highly interesting to include the above-mentioned TSPO KO model in future dual tracer PET studies with [^18^F]GE-180 and [^18^F]FET, all the more when investigating TSPO expression in brain tumors under therapeutic circumstances, e.g., after radiotherapy [17]. Pharmacological microglia depletion is another valuable approach for modulation of host TSPO levels, yet to be applied in experimental brain tumor models [51].

As in several comparable preliminary TSPO PET studies in GBM, a limitation of our study is the lack of resolving the contribution of specific cell types to the TSPO signal in direct correlation to in vivo imaging findings. Yet, we were able to show that [^18^F]GE-180 uptake in autoradiography and positive TSPO staining were congruent and co-localized with [^18^F]GE-180 uptake in PET. However, in order to clarify the source of TSPO signal at a cellular level, additional experimental methods such as immunohistochemical co-staining or innovative approaches of radiolabeled cell sorting [52] would be helpful, and such investigations are underway in preclinical brain tumor models. Some studies already aimed to quantify in vitro the respective contribution of different cell types to the overall TSPO signal, albeit with conflicting results: While some studies attribute most of the signal to neoplastic cells [45,53,54], others highlight a mutual contribution of several cell types [33] or rather claim a major role of GAMs in contributing to the overall TSPO signal in brain tumors [32]. This may in part be due to different experimental setups used in those studies. However, even within distinct studies, the range for the amount of TSPO-positive GAMs contributing to the overall TSPO cell population was high (e.g., ranging between 4.2% and 55%) [54]. This rather suggests that, on balance, the interplay of tumor and host cells in generating the TSPO signal in brain tumors is not yet sufficiently understood. In sum, TSPO PET imaging in glioma mouse models remains an exciting field harboring the perspective of better understanding the glioblastoma microenvironment at a molecular level.

## 5. Conclusions

[^18^F]GE-180 PET imaging facilitates non-invasive in vivo monitoring of TSPO expression in the GL261 GBM mouse model. [^18^F]GE-180 in vitro autoradiography is a convenient surrogate for ex vivo autoradiography, due to highly congruent tracer distribution in both methods, allowing for straightforward identification of suitable models and scan time-points on previously generated tissue sections for the design of future studies.

## Data Availability

The datasets used and/or analyzed during the current study are available from the corresponding author on reasonable request.
